# The contribution of coherence field theory to a model of consciousness: electric currents, EM fields, and EM radiation in the brain

**DOI:** 10.3389/fnhum.2022.1020105

**Published:** 2023-01-24

**Authors:** Eric Bond

**Affiliations:** Whitman College, Walla Walla, WA, United States

**Keywords:** coherence field theory (CFT), electromagnetic field, electromagnetic radiation, electric current, ebb effect, photonics, infrared, quantum entanglement

## Abstract

A paradigm in neuroscience is developing which views resonance as the phenomenon responsible for consciousness. Much progress is being made in the investigation of how resonance as oscillating flows within the brain’s electric field might result in production of mind from matter. But it’s mostly unknown how vibrations among features of matter such as nanoscale atomic structures and photonic waves may participate in forming the basic substance of first-person consciousness, meaning percepts such as colors, textures, sounds, thoughts, feelings et cetera. Initial evidence at the leading edge of quantum biology suggests that light and atoms combine to form synchronously resonating structures of contiguous energy which I have termed coherence fields. My hypothesis is that coherence fields as atomic nodes within expanses of integrating photonic waves are the fundamental unit of first-person percepts insofar as they arise from electromagnetic matter. A concept of quantum coherence is formulated based on a new phenomenology of matter’s nanoscale properties, and this is shown to tie what we have thus far discovered of neural anatomy into a comprehensive model of how electrical impulses travel through neurons as electron currents driven by coherence at the quantum scale. Transmembrane electric fields generated by ionic currents, synaptic phase regulation, and perhaps further mechanisms have been hypothesized as responsible for local field potentials (LFP) oscillations. Some insights into how emergent, macroscopic waves in the brain’s electric field may reciprocally impact LFP propagation to control arousal, attention, and volition are briefly discussed. Activation of neural tissue is closely linked to temperature variation, and it is hypothesized that this is not merely a waste byproduct but constitutes a signature of coherence field modulation, with photonic waves of a primarily infrared spectral range functioning as an interstitial medium of the basic percept field. A variety of possible routes to coherence field modulation are outlined that derive from the mechanisms of electric currents, EM fields, EM radiation, and entanglement. If future experimental designs continue to validate coherence field theory, this could set science on course to resolve the mind/body problem.

## Introduction: consciousness as the resonant structure of matter

One of the features that most defines brain matter is vibration: waves of electrical energy ranging from one millimeter to a dozen centimeters course through this organ (Alekseichuk et al., 2019), oscillating and flowing as an emergent property of neural networks. Most have some familiarity with the fact that these traveling waves are a primary signature of the arousal states such as thinking, imagining, remembering, focusing, etc., which we refer to as consciousness. EEG machines are of course employed to study the mind by observing the brain’s electric field. Vibration proves more fundamental than this, for all of the electromagnetic matter we sense *via* optical inspection, measure with instruments and scientifically model, is comprised of wavelike frequencies. We all know that light oscillates, with the distance between peaks of its superpositioned waveforms ranging from nanometers to kilometers. It is also common knowledge that electrons, as the particles which give atoms their shape, have wavelike characteristics that produce interference patterns upon contact with many surfaces (ER Services, n.d.a; Mairhofer and Passon, 2022). Vibration is so intrinsic to the structure of matter that a paradigm in the scientific study of consciousness’ substance is materializing which seeks to model it in terms of resonance, for example, Hunt and Schooler’s General Resonance Theory (GRT). This theoretical approach anticipates that vibrations in matter are not merely a signature of consciousness but rather the essence of the mind itself.

In theorizing consciousness, GRT has so far focused on models of the brain’s electric field, most basically reducible to LFPs (local field potentials) as oscillative perturbations at the scale of individual neurons. Electric field oscillations superposition to produce emergent contours we know as brain waves and GRT is committed to closely examining patterns of electrical energy as they arise in different combinations and locations within the brain, for they may be the actual substance of experience. The present article tries to base GRT’s brain wave account of consciousness on lower, quantum levels. Neuroscience is in the beginning stages of determining the extent to which the wavelike behavior of matter at the quantum scale is relevant for the modeling of sensations, perceptions, and stream of consciousness. It will be proposed here that key mechanisms of the first-person mind fall within the purview of what has traditionally been the quantum physics’ domain. The goal of this article is to investigate some ways that phenomena operative at the nanoscale may participate in generating macroscale phenomena including brain waves and minds.

Historically, quantum mechanics has tended towards formulas and mathematization, with the motto being “shut up and calculate” while phenomenological considerations were mostly neglected. This is for an obvious reason: it is so difficult to directly observe matter at the nanoscale that even the most introductory progress in this area was untenable. But recent discoveries in the realm of cellular anatomy have changed the situation, and this article will attempt to elucidate how neural structure in particular sheds light on the qualities and roles of quantum-scaled phenomena in biological systems.

The article starts by giving a description as to why the concept of a matter field is central in the quest for a model of consciousness, then some background into why quantum mechanics as the foremost determinant of this concept relies so heavily on models of statistical probability rather than physical structure in formulating its models. Despite the discipline’s antirealist leanings and ambiguity in how its quantitative abstractions are to be interpreted, some key realist insights are possible which provide for a general definition of what will be delineated and explained as quantum coherence. This concept of coherence is a powerful idea, for it makes sense of all the empirically derived facts thus far disclosed about a neuron’s component structures, allowing us to arrive at a much deeper comprehension of neural function, from the subatomic to cellular scale. Essentially, the flow of electrical energy around an aqueous solution inside a neuron is driven by differentials in electron density caused by ion diffusion, which seems to be why ion channels are positioned where they are in the cell and how signals are transmitted intraneurally. Hypotheses are discussed in regards to how LFPs might emerge from either dynamics of the synapse, or electron and ion currents abiding by the newly outlined principles of quantum coherence. LFPs in turn synchronize into emergent waves of oscillation with phase distributions linked to attention, awareness, and volition.

An empirically backed hypothesis will be made that electromagnetic radiation and molecular arrays jointly oscillate in what I call a coherence field, the signature of which is vibration measured as temperature variation. Temperature changes occur when neural networks activate, suggesting that cognitive processes are tied to thermal energy, consisting in the vibration of biochemical structures within photonic fields typically centered on infrared portions of the EM radiation spectrum which effectively penetrate the aqueous solutions within and between neurons. It is proposed that this complex of particle vibrations and radiative waves is not merely the waste byproduct of neuron firing and chemical reactions but rather a vital component of mechanisms binding electromagnetic brain matter into the substance of perception. Relatively nonlocal mechanisms of consciousness are of course in effect as an additional factor, but if electrical coherence currents, EM fields, and EM radiation coordinate with molecular complexes as the electromagnetic facet of coherence field structure, a resultant coherence field theory (CFT) may enable the GRT framework to begin discerning how the nanoscale of quantum physics renders matter a perceptual field.

## Consciousness, the brain, and quantum coherence

One of the main topics that arise in consciousness theory is the binding problem: how can trillions of atoms and billions of cells participate in producing the more or less integrated medium of awareness we introspect? The body and brain are intimately involved in generating this experiential substrate, for awareness seems to largely extinguish when physiological processes cease, but it is not easy to discern how the holism of conscious experience inheres within brain matter and is in large measure instantiated by it.

As a parallel investigation, 20th and 21st-century physics have come to rely on the concept of a field: matter is not fundamentally solid and stable, but rather a vast array of ripples or disturbances in a sort of fluid medium characterized by perpetual motion, with relatively persistent focal points of perturbation being what we observe and model in the form of particles (Strassler, n.d.). As loci of energetic perturbation, particles radiate causality farther than characteristic densities we directly measure using mass, through a spatially extended substrate we do not yet fully grasp. The concept of matter as a field of fluctuating, flowing perturbations and the concept of experience as a stream of consciousness which contiguously saturates our reality is intuitive to analogize. But constructing a viable model must conjoin domains of inquiry that diverge widely in content and methodology. How can the sciences of perception, personality, and meaning be reconciled with the sciences of matter with their fundamental reliance on the modeling of unconscious mechanisms? If any synthesis is to be had, it seems destined to initiate by explaining brain processes as a physical field, and examination of the organ’s wavelike, diffusive properties is moving neuroscience in that direction. The aim is to explore some hypotheses which may further the modeling of the brain’s coordination with experience as a physical field.

A detailed history of the physical field concept is beyond this article’s scope, but suffice to say that our most precise experiments and calculations reveal the continuum of matter as divisible into basic quanta with measures such as an almost infinitesimally small unit of distance called the Planck length: 1.6^*^10^−35^ m. To give a sense of the scale, protons are about 100 million trillion times larger. This quantity originated at the turn of the 20th century as a calculational tool that integrated quantum, gravitational, and eventually relativistic units of measurement while lacking much physical meaning, though modern string theory is a prominent attempt at theoretically modeling this scale (University of South Wales, n.d.). However, a related term called Planck’s constant (approx. 6.6^*^10^−34^ joule-seconds) correlates the frequency of EM radiation with its wavelength and is a foundational component of quantum mechanics (TechTarget, n.d.), allowing physicists to probe, model, and technologize electromagnetic matter by observing how atomic and subatomic particles quantize frequencies and corresponding wavelengths of EM radiation while interacting with them.

During the inception of quantum mechanics, it was confirmed by experiments which created interference patterns by scattering electrons from crystals that these particles have wavelike properties. Louis de Broglie developed a theory based around arranging circular, wavelike electron “orbitals” according to a constrained range of oscillative shapes characterized by quantized ratios where constructive interference obtains, similar to how a plucked guitar string vibrates in whole number ratios of its length (ER Services, n.d.a). But when EM radiation was emitted into atoms experimentally, scientists found that each individual trial produced a more particulate than a wavelike signature in a different region within the atom. Furthermore, this location could not be predicted exactly from trial to trial because the higher the frequency or energy of EM radiation, the more it knocked the electron out of its natural trajectory, altering the momentum, and the lower the energy, the somewhat less particulate an electron registered upon contact, making its position less exact. The fundamental imprecision of these measurements was quantified and codified as the Heisenberg uncertainty principle. By contrast, hundreds of trials resulted in a probability distribution of more and less likely locations that looked like a cloud of particulate density, and the shape of this cloud could be reproduced with great precision (ER Services, n.d.b). Mathematical tools were fashioned for performing calculations on these probability distributions, namely Heisenberg’s matrix mechanics along with Schrodinger’s wave function (Casado, 2008), and quantum mechanics remains fundamentally probabilistic to this day, even in its most high-tech applications.

**Figure 1:**
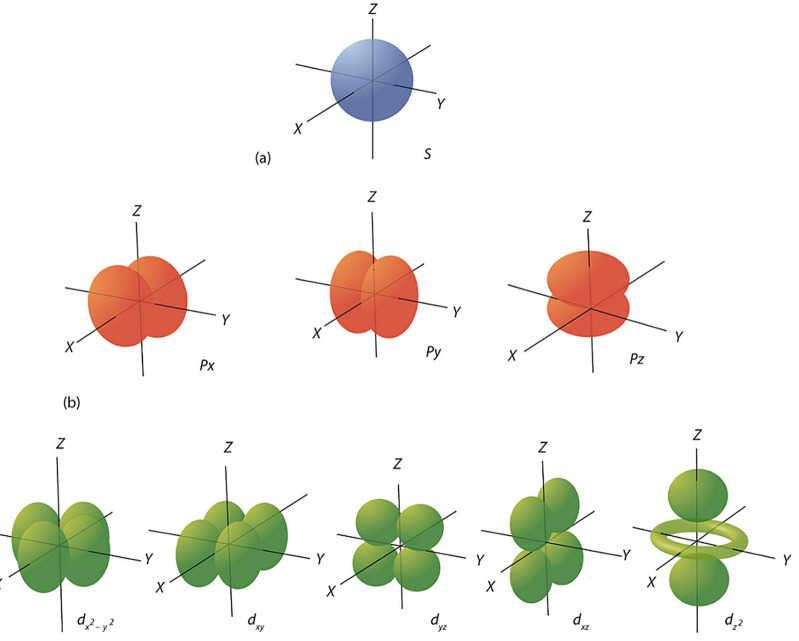
Diagram of atomic s, p, d orbitals (Blendspace, n.d.).

So basic understanding of matter is founded on relative probability, with the textbook image of how electrons are arranged in atoms depicted by squaring Schrodinger’s wave function to enable a geometry of probability density (Morin, n.d.). These geometries are assumed to be three-dimensional for reasons of clarity, superimposed on an “x, y, z” coordinate system in ways that maximize symmetry of charge since negative charges repel (Dill, 2008). The shapes thus formed include spheres, dumbbells, and doughnuts, in all sorts of hybrids (Figure 1).

Quantum mechanics is one of the most accurate models in science, matching the results of thousands of experiments to impeccable precision, but is nonetheless an approximation, and uncertainty persists about what is going on beneath the superimposed math. The crux of the dilemma is how a greater than zero probability exists for a particle such as an electron to be anywhere (saylordotorg.github.io, n.d.) while we experience matter as localized to particular regions of space. The math says that every particle is to some extent everywhere at once as a universal superposition of states, while real particles reside at a particular place and time, so what is the actual state of the matter itself when what we quantify is so different from what we intuit?

Competing interpretations of quantum mechanics have been proposed which fit the math equally well, though experiments are beginning to achieve the capacity to adjudicate between them. The many-worlds interpretation hypothesizes that a particle splits into multiple, largely noninteracting timelines when undergoing certain types of perturbation such as measurement so that superposition is undissolved by factors of localization like particle collisions even though most of these superpositions are not to this point scientifically observed. The pilot-wave interpretation assumes that particles such as electrons are guided along trajectories by underlying wave perturbations which have not been witnessed directly. Spontaneous localization interpretations attempt to model physical matter as pockets of locality that form within the probability plenum in a phenomenon directly proportional to the quantity of perturbation, and a host of different parameters for how this localization occurs have been fashioned with the aim of fitting experimental data. But enough doubt remains that the traditional Copenhagen interpretation is the most popular, simply asserting the math should be viewed as working agnosticism, a technique allowing us to predict the relationship between initial and final probabilities of a material system without telling us anything realist about causality (Mohanmurthy, 2020).

Despite the incertitude, some rudimentary realist knowledge can presently be gleaned from the probability model that is sufficient for the purposes of neuroscience. First of all, though a probability exists for the energy of every particle to be anywhere, each particle involves a range of most to least likely locations that eventually declines dramatically as one strays from the center of mass, and reductions in probability correspond to a diminishment of energy density, meaning regions equidistant between centers of mass tend to be less energetically dense (saylordotorg.github.io, n.d.). So centers of mass are various forms of energy maxima, and equidistance between them relative minima, a principle seeming to apply all around us, from electron orbitals, to atoms, planets, etc. We also know, at least insofar as electromagnetic properties obtain, less mass or energy density corresponds to more propensity for energy to flow through that region of space. For instance, the less dense that electrical energy is at a specific location, the more rapidly this energy can accelerate. Atom on atom causality of an EM field, provisional of maximum diffuseness (density minimum) when atoms involved are not chemically bonded, almost instantaneously reaches or nears the typical max speed of magnetism and light, 300 million m/s. Currents comprised of electrons which are density maximums within the EM field of an atom can attain 90% of the speed of light in a copper wire due to a cascade of local displacements called signal velocity which travels along its length, but rarely any speed in excess of that, especially at the micrometer scale or larger. Many electric currents, again a directional flow among adjacent density maximums, reach average signal velocities that can be closer to 50% of the speed of light. This is a consequence of the idiosyncrasies in specific atomic structures along with a material system’s entropy, the amount of disorder from factors such as temperature that increase local agitation, preventing electrons from synchronizing within relatively large spaces (Bond, 2022a).

Under conditions where electric currents cannot flow micrometers or larger distances because of entropy, electromagnetic motion tends to commove haphazardly and settle into maximum average locality, a state which has been termed decoherence. When conditions are such that electrical energy flows synchronously, this is a state of coherence. So a spectrum of relatively decoherent to relatively coherent states exists among electromagnetic matter. An atom’s electron orbitals or density maximums in and of themselves are relatively coherent, to the extent that atoms can be modeled as individual units of superpositioned probability waves. Trillions of atoms jostle entropically enough in typical Earth environments that relative decoherence prevails and net motion is modelable in terms of classical space and time. Chemical bonds range between a maximally decoherent and maximally coherent state, as a sort of short-ranged coherence at the boundary of Newtonian and atomic structure. And electric currents constitute a special case where atoms are induced to engage in macroscopic coherence transcending the baseline boundaries between microatomic and macroatomic (Bond, 2022b). Electricity is made to flow by charge differentials in the matter, with greater charge differential (voltage) as a general rule causing more rapidly accelerating currents (amperes). It will be shown that the most plausible model for signal transmission in a neuron is derived from these coherence principles.

## Electric coherence currents and EM fields within the brain

**Figure 2:**
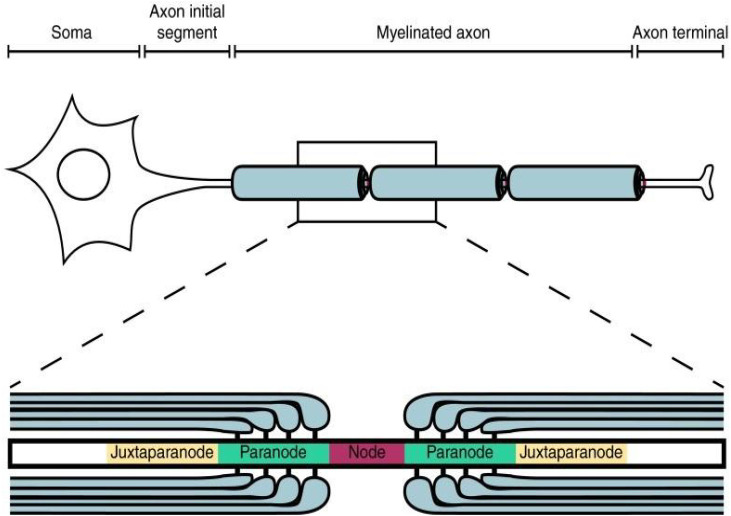
Nodal, paranodal, and juxtaparanodal regions (Arancibia-Carcamo and Attwell, 2014).

It is well-established that neural signaling is modulated by the diffusion of ions through channels in a neuron’s membrane, but ion collisions cannot explain some features of signal transmission. Researchers have discovered that each node of Ranvier, where voltage-gated Na^+^ channels let Na^+^ into an axon, is flanked by paranodes, where the myelin sheath attaches to the outer membrane, and these are flanked by juxtaparanodes, where voltage-gated K^+^ channels are located that let K^+^ flow out of the cell when open (Figure 2; Arancibia-Carcamo and Attwell, 2014). Ion diffusion provides no reason for voltage-gated K^+^ channels to be strategically placed at the juxtaparanodes. In theory, larger diameter axons involve less axial (lengthwise) resistance due to greater volume and more dilute ion concentrations. This would allow more rapid axial diffusion rates, necessitating that nodes of Ranvier be farther apart so as to keep signal strength the same, but nodes of Ranvier are actually spaced closer together in larger diameter neurons (Ford et al., 2015). Computer simulations demonstrate that widening nodes of Ranvier slightly to significantly increase the quantity of voltage-gated Na^+^ channels does not increase the rate of signal transmission with more ion diffusion (Arancibia-Carcamo et al., 2017). And a neuron’s signal can of course travel meters in milliseconds, far exceeding the rate of diffusion. Where a description based on ion diffusion alone falls short, applying the idea of electrical coherence current succeeds. The coherence model has not at this stage surpassed the status of the Gedanken experiment, but ties all we know about the chemistry and anatomy of neurons into a complete picture so is deserving of concerted empirical investigation.

The solution internal to a neuron is made up primarily of water molecules and positive ions. H_2_O is of course a polar molecule, its hydrogen atoms being the positive poles and the oxygen atom a negative pole, bent at the fulcrum. A nanoscale solvation shell forms around each positive ion, with negative poles facing inward and positive poles outward. Thus, the cellular solution contains a complex contour of positive and negative charge. Since positive ions lack an electron, the electromagnetic density of aqueous solution at their locations would be reduced. Asymmetries in electron density perpetually shift positive ions and water molecules around in pursuit of equilibrium, a nanoscale agitation which causes the solution to on average be maximally decoherent as its baseline condition.

When Na^+^ floods into the axon at a node of Ranvier during an action potential, electron density decreases in that region. This creates a positive terminal that induces an electric current to flow towards the node, but the current begins adjacent to the node and cascades outward into successively distant regions. Because propagation slows due to electron mass inertia when charge is constant, I have named this the “ebb effect”. The ebb effect has not been verified by experiment but should be observable within any aqueous solution of ions that contains regions of both charge differential and uniform average charge.

The electron density of atoms is enveloped in an EM field that acts remotely, perturbing at or near the speed of light as atoms move. When an electrical coherence current initiates, the leading edge of procession away from the node is accompanied by an EM field fluctuation, probably the trigger by which depolarization activates voltage-gated ion channels, *via* a temporary nanoscale magnetism caused by synchrony of electric current flow.

Electric current initialization decelerates through the paranodal region, and upon reaching the juxtaparanode its field perturbation triggers voltage-gated K^+^ channels to open and let this ion rush out of the axon. The spike in electron density propels current through internodal space at a significant fraction of light speed despite resumed slowing. The motion of this phenomenon is complex, depending on local ion concentrations, positions, and the relative rate of electron vs. ion flow, but thought experiments preliminarily suggest that electrical energy might saturate at the node of Ranvier due to a signal velocity’s relative rapidity. This would form a sort of electron wall so that greater electron density can only travel towards internodal space. Voltage-gated K^+^ channels then serve to greatly increase electron density by vacating positive ions from the juxtaparanode, at a faster rate than Na^+^ influx. A substantive breadth of higher electron density thus materializes near-instantaneously at the juxtaparanodal and paranodal regions, causing pressure which is released by the flow of electric current through internodal space and to the next node of Ranvier. An accompanying field may trigger the intervening, downstream juxtaparanode to depolarize, while the subsequent node of Ranvier has usually not been completely repolarized, and charge differential accelerates current towards the node of Ranvier. EM field stimulation then causes voltage-gated Na^+^ channels to let this ion flow into the axon, a chain reaction that continues to the axon terminal where a synapse occurs.

An increase in electron density at the downstream juxtaparanode and paranode would also induce current to flow in the upstream direction, back into internodal space. It is at least conceivable that an alternating current of concentrated electron density could cause a sort of reverberation within internodal space which might be a fundamental aspect of the mechanism responsible for electric field oscillation. This may be supported by recent research improving the resolution of LFP structure using a Discrete Pade Transform (DPT) analysis. The technique revealed low amplitude, high frequency, irregular harmonics that comprise 90%–99% of the total quantity of frequencies (Perotti et al., 2019), a noise component perhaps hinting at ultrafast, intracellular reverberations within the LFP, but a definitive link between the present thought experiment and DPT data awaits further study. The extent to which these mechanisms are possible in dendrites is also empirically uncertain.

Dendrites have clustered Na^+^ channels as well, so an EPSP (excitatory postsynaptic potential) takes place *via* at least the ebb effect mechanism. Cl^−^ channels are located at the dendrite/soma junctions to halt EPSPs with a Cl^−^ influx that initiates current traveling upstream into a dendrite, from greater, negative electron density to lesser, positive electron density. This current is called an IPSP (inhibitory postsynaptic potential). When Cl^−^ influx and IPSPs wane, with EPSPs cumulatively strong enough to breach the soma *via* the ebb effect, a threshold is crossed, probably abetted by the subsequent resumption of Cl^−^ influx, and this relatively large electron density accelerates rapidly towards the greatest quantity of voltage-gated Na^+^ channels and Na^+^ ions in a neuron at the axon hillock. K^+^ leakage channels are present throughout the outer membrane to sustain positive ion concentrations as a kind of electrochemical chassis allowing ebb effect flow to trump decoherence effects, which are more substantial when greater amounts of water agitate the solution locally due to denser polarity. Sodium-potassium pumps help maintain diffusion gradients across the membrane by a constant ferrying of two K^+^ ions into the cell accompanied by three Na^+^ ions out of the cell.

Microscopic platinum sensors have been inserted into individual neurons, revealing a crystalline structure located just beneath the axon’s outer membrane, wrapped around a core support framework of microtubules (Bandyopadhyay, 2022). This probably assists in holding ion concentrations at levels provisional of the ebb effect. A greater volume-to-surface area ratio may surround this structure in larger diameter neurons, necessitating that nodes be spaced closer together to compensate for dilution effects and a consequently less powerful electron current due to more resistance from decoherence.

So based on what we know of cellular anatomy, an explanation for signal transmission in neurons which appeals exclusively to ion diffusion and transport is unsatisfactory, but the concept of electric coherence currents traveling through a chassis of positive ions at significant fractions of light speed meets all current requirements for a successful model, though experimental verification remains to be performed. How then does a flow of ions and electricity associated with individual neurons result in macroscopic oscillations of the brain’s electric field, and does this field have some functional role in consciousness’ architecture?

Wave phases of individual neurons coordinate as supracellular electric field oscillations in a process termed “phase-locking”. These electric fields of more or less in-phase neural networks then constitute emergent flow shapes which reciprocally impact the firing of individual neurons. Transcranial magnetic stimulation by electric fields having properties resemblant of the organ’s endogenous field (Frohlich, 2014) as well as the application of similar fields to *in vivo* and *in vitro* preparations of neural tissue (Frohlich and McCormick, 2010) demonstrate this ultrasynchronizing entrainment effect. Phase-locking’s mechanism is still a mystery, but progress is being made.

Preliminary research suggested a neuron’s lipid membrane almost fully absorbs an electric field produced by the internal electron current so that it only extends a few nanometers beyond the membrane’s surface (Anastassiou et al., 2011). This would seem to imply the current is not directly involved in phase-locking. However, recent research has provided evidence that ephaptic coupling occurs at much greater ranges as an important factor in communication between neurons. Slow wave oscillations of the mouse hippocampus, less than 1 Hz, were proven to synchronize neural activity in slices separated by as much as a 400 μm gap, which eliminated synaptic transmission and gap junctions as variables. The entrainment effect propagated at 0.1 m/s, too fast to be accounted for by ion diffusion. An anti-electric field blocked this phenomenon, adding evidence that ephaptic coupling is the mechanism (Chiang et al., 2019). Further study revealed pharmacological blockers to be incapable of inhibiting synchrony while stimulating intact slices using an electric field with similar properties to the tissue’s endogenous field induced a self-propagating wave of comparable nature. Applying a voltage clamp completely blocked synchronization, still more evidence that ephaptic coupling is the mechanism (Shivacharan et al., 2019).

It is not yet entirely clear how and to what general extent ephaptic coupling is active in conjunction with biochemical features of the neuron, but a theory has been proposed. Researcher Colin Hales developed a computer model suggesting the global, static field that pervades neural membranes of the brain is accompanied during neuron firing by fields arising from ion channels operating both individually and in tandem. He postulates that these overlapping electric fields caused by ions moving more or less coherently through channels densely concentrated within a neuron’s membrane, flowing at a rate similar to electric current in a copper wire, 90% the speed of light, generate the transmembrane impact upon nearby neurons revealed by experiment. When certain parameters are introduced to this model, the most significant being sufficient synapse-mediated synchrony among neural networks, then ion channel fields projected beyond the neurons giving rise to them modify firing thresholds into a collective form, tightly binding groups of neurons as phase-locking’s mechanism (Hales, 2014).

It seems plausible to the present author that a combination of K^+^ leakage channels and sodium-potassium pumps positioned throughout the neural membrane could produce a transmembrane electric field extending the full length of a neuron *via* constant flurries of ion transport, binding adjacent cells into relatively stable superstructures through the mutual influence of their fields. Holistic activation of the voltage-gated ion channels at each node by lengthwise coherence currents traveling at a sizable fraction of light speed would then cause surges of transmembrane electric field behavior, a further influence inducing clusters of neurons to fire in unison. So though electric field oscillations of a neuron may at the base be the consequence of intracellular force exacted by electron density disequilibration and resultant lengthwise flow, these currents might be synced into phase-locked, more or less in-phase conglomerates by ionic currents transiting through channels, a dual mechanism of electrical energy from different sources that induces emergent electric field patterns which stimulate collective firing. Intracellular electron currents might evince irregular microreverberations in the field, K^+^ leakage channels and sodium-potassium pumps a constant, low-level field noise from somewhat loosely synchronized populations of ionic current (which might also contribute to the irregular harmonics of DPT), and nodal fields the more regularized oscillatory patterns of LFPs. Research is ongoing into the origin of neural oscillation, and we will know more about how and why this phenomenon occurs in the coming years. As in the case of coherence currents, an ion channel hypothesis requires more empirical validation.

Evidence is accumulating which suggests that at least some synapses do not transition between inactivated and activated states as a continuum correlated with the gradualized flow of thousands of molecules and ions, but rather snap into three or more discrete states linked to the degree of synchronous potentiation. Interestingly, a model of this phenomenon has shown that at certain frequencies of neuron firing and rates in the transition between discrete states, oscillations of a presynaptic and postsynaptic neuron can be in-phase, so phase-locking may be mediated by synaptic synchronization (Abarbanel et al., 2005).

It is apparent that coherence currents induce transmembrane LFPs (local field potentials), hypothetically phase-locked by mutual projection from ion channels, synaptic synchronization and/or alternate mechanisms. Emergent oscillation and flow shapes in the brain’s electric field, of the kind EEG distinguishes from those of individual neurons, may then magnetically orchestrate flurries of molecular machinery, similar to how electric currents drive the operation of appliances by exacting organized magnetic effects upon their structure. Actually, brain cells may be more akin to an ecosystem that is especially fine-tuned in comparison to most physiology, with components fluxing in holistic ways partially under their own power while tightly knit by varying EM field stimulation, a cross between mechanism, food chain, and mass migration. It seems probable that brain waves are more than an epiphenomenon, flowing through neural tissue to participate in morphing swaths of molecular structure into simultaneity. The more phase-locking an electric field attains among neural networks, the more large-scale, unified, and self-directed its functioning can be. Research indicates that the behavior of the brain’s electric field consists ofhing, that it have regionally linked oscillation patterns as well as a 40 Hz signature corresponding to individual neurons, superimposed on slow wave oscillations emergent from the whole brain, with a large, roving concentration of semi-stable gamma activity which blends with local oscillations while it moves. This drifting density of macroscopic integration could be the primary orchestrating factor in experiential awareness (Hunt and Schooler, 2019). It could also be a root of volition as proposed by CEMI (conscious electromagnetic information) theory (McFadden, 2020, 2021).

The coherence current model and some auxiliary concepts seem to put certain basic principles of the mind’s organization insofar as it connects to the brain’s electromagnetism within reach, but we still lack the total picture, for this does not in itself necessitate that consciousness look or feel like anything, that it has features of awareness as opposed to being machinery, a mere technological gadget. How do percepts arise in conjunction with physiology of the brain and body?

## EM radiation as a binding agent for the physiological substance of perception

All EM fields are filled by a vast array of undulations which readily superposition while flowing between and in synchrony with atoms, what we know as EM radiation or light. EM radiation can conceivably constitute the interstitial texture of perception’s substance, so the question then is how to characterize the properties of this light energy. Electrons as electromagnetic constituents of massive atoms, the density maximums, and light as textural substantiality between atoms, the density minimum, evince a counterintuitive property known as entanglement. Entanglement is a process by which particle states such as spin in electrons or phase in photons correlate across distances at faster-than-light speed. It occurs *via* relatively nonlocal forces that are still poorly understood, which underlie coherence in all its forms, more fundamental than electromagnetism (Franson, n.d.). In relatively diffuse, minimally entropic, or relatively homogeneous material structures such as gases of more or less minimized temperature and simple chemical composition (Irving, 2020), faster than light entanglement can readily take effect, but very exacting conditions must be generated for the phenomenon to presently be observed in the lab. Under more common circumstances such as the flow of electric current through a compact structure such as a metal, or through an entropic substance such as an aqueous solution, or through heterogeneous matter such as an organic body, the nonlocality of coherence is dissipated by the medium’s baseline decoherent state so that rates slower than the speed of light obtain (Bond, 2022a, b). Coherence among electromagnetic particles of substantial mass thus tends to be mitigated in various degrees by density, a sort of rate bottleneck effect more pronounced the greater the complexity of density contour.

EM radiation, by contrast, is much less massive and does not have nearly the same constraints as electrons or atoms. Congregates of photons can evince statistically significant entanglement correlations across distances of at least 15 km (Filmer, 2013). Light has further properties unique for electromagnetic matter, filling nonvacuum spaces populated by atomic structure as a wave, and much more readily superpositioning into additive structures than atoms, put on full display by the wide range of wavelength combinations associated with the visible spectrum. EM fields are made to undulate as EM radiation when electrons in atoms or electric currents accelerate or decelerate, and most electromagnetic matter does to some extent, so nature is saturated with light (Northwestern, n.d.). This light interacts with atoms in complex ways that are still rudimentarily understood, but we know for sure that its wavelengths can blend into atoms when energy is complementary. Many photons scatter as they collide with atoms, a phenomenon known as the Compton effect, but light also forms vibrational complexes of atomic nodes within photonic fields (Dill, 2008). Radiative/molecular superpositions as synchronously vibrating arrays of electromagnetic matter are an excellent candidate for the substance of percepts, and research into the connection between photonics and awareness is showing promise.

In the initial analysis of light’s interaction with biological systems, it was discovered that photosynthetic reaction center complexes achieve 100% energy yield from UV radiation because light waves take multiple routes or flow through numerous chlorophyl molecules as they are translated into chemical energy, fully absorbed by a reaction center hub without fail (McFadden, 2014). Chlorophyl arrays are such that EM radiation blends into them like they are a pool of water and photons a bead of this water, conjoining as a coherent energy field. Early research into the response of neurons to light exposed them to the visible and UV spectrum. It was found that this relatively high energy EM radiation affects neural function, but primarily due to the degradation of ion channels and additional structures, reducing synaptic efficiency (Khoshakhlagh et al., 2019). Subsequent examination has proved more auspicious, however.

A long-standing hypothesis about the source of consciousness, Roger Penrose and Stuart Hameroff’s Orch-Or (orchestrated-objective reduction) theory, proposes that microtubules are compact enough in the brain to produce a wide array of pulsing superpositions responsible for awareness (Hameroff, 1998). The model has faced criticisms from scientists who claim the brain is too hot and wet to support the coherence of this kind, but recent experiments have aimed to assess whether light induces a coherent energy field in microtubules where molecular structure alone cannot.

Microtubules contain light-sensitive amino acids such as tryptophan, and the absorption of UV light was recently tested. A solution of microtubule fragments exposed to UV light was proven conducive to remote energy transfer between component tryptophan molecules. Anesthetics inhibited the phenomenon, hinting at a link with consciousness. Combining this data with a model of tryptophan positioning inside intact microtubules suggested that the amino acid can mediate a coherent energy field spanning the microtubule’s entire length, ranging to 50 μm. The only source of UV light in a typical cell was hypothesized as perhaps the oxidation reactions of mitochondria, so it is doubtful these wavelengths have much of a functional role in the brain, but it becomes increasingly apparent that light superpositions and entangles among relatively large molecular structures to produce coherent energy fields in a wide range of circumstances (McIver et al., 2022; Neven et al., 2022). So the question is whether some alternative light source exists within the brain to cause an expansive energy coherence.

An obvious option for endogenous light in the brain is infrared radiation, which saturates physiological structures while constantly absorbed and emitted by rotating and vibrating atomic bonds. The capacity of the infrared spectrum to transmit through aqueous solution quickly diminishes as this radiation’s wavelength increases from 1 to 10 μm, but plenty of circumstantial evidence ties the thermal energy of molecular motion associated with infrared radiation, better known as temperature, to brain function. Brain tissue temperatures have been measured to exceed those of the blood by 0.5°C–0.6°C in various mammals. In rats, the temperature of the hippocampus increases 1.5°C–38°C when actively exploring. In male finches, temperature of brain tissue increases during variance in song tempo. Feeding and social interaction produce rapid, unique, and relatively long-lasting brain temperature elevations, occurring faster and with greater magnitude than those of the arterial blood supply. In humans, somatosensory cortex temperature increases during nerve stimulation, and likewise for motor cortex and bodily movement. Many brain regions such as the substantia nigra alter their activity when the temperature is varied. The rise in temperature of neuronal pathways is generally linked with sensory stimuli, and correlations between temperature and data obtained on resting potential, action potential, nerve conduction velocity, and synaptic transmission are well-established. Anesthesia lowers brain temperature, a sign that infrared radiation may be linked to conscious awareness. The total brain varies in temperature by 1°C–3°C in some animal models. The relationship is obvious, but whether temperature contributes some function or is merely a byproduct remains uncertain. Indications exist, however, that neurons may be tailored for the purpose of sustaining the brain’s infrared spectrum at robust levels. A rapid spike in temperature of two degrees microCelsius occurs during action potentials, hinting at a general connection between nerve firing and a boost to the infrared spectrum (Wang et al., 2014). So if we hypothesize that neurons are designed to expand the quantity of infrared light while regulating its local behavior, how might this mechanism work?

Assuming the coherence flow model is accurate, as it certainly seems to be, lengthwise signals are transmitted through a neuron as electric currents which attain a relativistically significant percentage of light speed, so the mass of this rapidly moving matter increases. Experiments in the first half of the 20th century suggested that relativistic mass has an underlying physical cause, while many modern approaches incline to view relativistic mass as a conceptual tool to be dispensed with at will (Gibbs et al., 2012). Debate rages, but regardless of the real source for theoretical mass increase when transitioning to high-velocity states, some empirically based conclusions of a rather simple nature can be drawn insofar as light emission correlates with relativistic momentum in electrons. We know from technological applications that matter moving at relativistic speeds emits higher energy (frequency), shorter wavelength EM radiation while it decelerates, and lower energy, longer wavelength radiation while it accelerates. For instance, when a beam of electrons traveling at half the speed of light collides with a metal plate in an x-ray machine, it emits high energy braking radiation in the x-ray portion of the spectrum (Arpansa, n.d.), and accelerating current in a radio antenna emits low energy radio waves (Astro, n.d.). Essentially, if an accelerating coherence current is almost instantaneously compressed as it alternates, EM waves will be emitted proportional to speed, total size, and perhaps lesser overall density of the current (in addition to waves at further spectral ranges), and if a decelerating coherence current is likewise compressed, EM waves are emitted in proportion to speed, size, and perhaps greater density of the current segment that is decelerating. So if current acceleration is sustained in a neuron, the spectrum of EM radiation will be prone to lengthen, and the reverse is true for decelerating current, with the quantity of radiation increasing in both cases.

During an action potential, the electric current accelerates between a node of Ranvier and adjacent juxtaparanodes, while gradually decelerating as it traverses internodal space. If this current alternates multiple times between juxtaparanodes following an action potential while changing velocity it might be possible to generate a photonic field. But it is unclear how sustained this field would be between action potentials or whether biochemistry is diverse enough in the axon, a structure probably tailored for long-range signaling at the expense of complex intracellular machinery, to generate a photonic/molecular field comprised of rich assortments of wavelength. Additionally, myelin encasing the axon would likely tend to reflect this radiation, preventing it from exacting multicellular effects.

At the synaptic junction, the current accelerates from single positive ion concentrations (Na^+^ and K^+^) at the last node in the action potential chain to a lesser electron density of Ca^2+^ concentrations near the axon terminal. Current would also accelerate from the first node in a dendrite to its upstream tip, on the opposite side of a synapse. In order for acceleration to be sustained, Ca^2+^ would have to cycle into and out of a neuron at rapid rates, continuously drawing energy away from nodes with a replenishing supply of lower electron density ions. Indications are that ions travel through ion channels *via* quantum mechanisms, again at approximately 90% light speed, so the cycle might be near-instantaneous enough to hold acceleration stable. But at present, more research into neuron anatomy near the synaptic junction is necessary before this hypothesis can be corroborated or refuted.

It seems more feasible at this stage to postulate a model for current acceleration in the soma. A tapering from more to less positive ion concentration is maintained between the largest quantity of Na^+^ channels and ions in a neuron at the axon hillock and relatively expansive space of the soma with its lesser rate of Na^+^ and K^+^ reuptake. This tapering ranges all the way to cellular space near the dendrite/soma junctions, where Cl^−^ channels and ions maintain a much higher electron density. Cl^−^ influx during an IPSP blocks EPSPs from propagating into the soma, followed by some Cl^−^ reuptake and an accompanying diminution of the IPSP. When the IPSP wanes, the ebb effect of EPSPs can draw greater electron density around the base of dendrites out of more interior regions of the soma. This is likely combined with a well-timed renewal of Cl^−^ influx such that electron density increases slightly while simultaneously breaching the positive ion gradient. Once this greater electron density reaches the axon hillock’s sphere of influence extending far into the soma, it accelerates rapidly towards the axon hillock. Upon reaching the axon hillock, a companion EM field fluctuation triggers large quantities of Na^+^ to rush in, sustaining acceleration from the opposite side due to greatly reduced electron density even as the relatively negative charge initiated at the dendrite/soma junction reaches a minimum due to dilution. As Na^+^ ions again diffuse into the soma, the gradient of positive charge is replenished, and though the overall strength and influence of positive charge lessens in the soma, Cl^−^ concentrations increase and regain a maximum, driving acceleration from the opposite side.

To summarize:

At the dendrite/soma junctions:

1. Cl^−^ influx, concentration, and electron density maximum
2. Cl^−^ concentration and electron density attenuation
3. The ebb effect force of dendritic potentials followed by resumption of Cl^−^ influx
4. Electron density from Cl^−^ concentration at a minimum, with continued influx

Instigated by the axon hillock:

1. Na^+^ concentration attenuation
2. Greater Na^+^ concentration attenuation
3. Na^+^ concentration minimum
4. Na^+^ influx and concentration maximum

Thus, a flux of Cl^−^ concentration maximum to minimum coupled with Na^+^ concentration minimum to maximum conceivably maintains a constant acceleration of electric current through the soma. As in the case of possible current acceleration around the synaptic junction, this model needs empirical verification.

**Figure 3:**
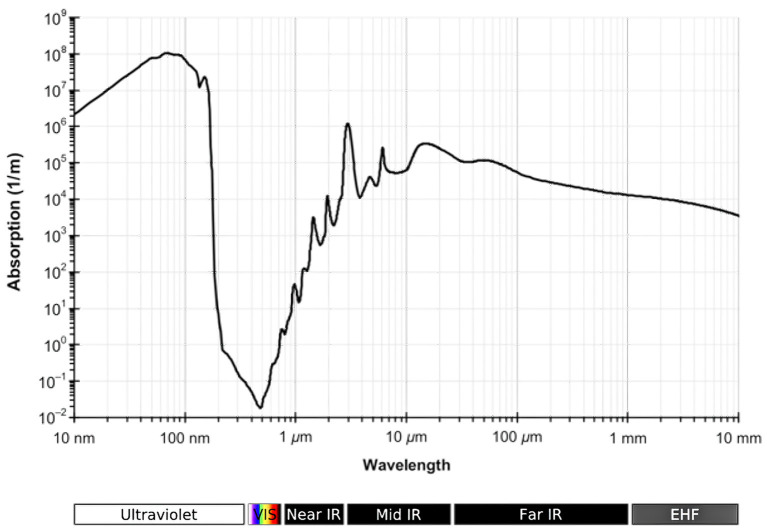
Absorption spectrum of liquid water (Kebes 2008).

So if current continuously accelerates at the synaptic junction and within the soma, what would be the properties of emitted EM radiation? Applying the nascent but plausible concept of relativistic current presented in this article, neural currents have no circuit to stabilize their velocity as in electrical wiring, so if the charge is constant they would probably initiate at the same rate as baseline agitation from decoherence and decelerate due to the ebb effect. EM wavelengths produced then hover at around 1 μm, slightly longer than the boundary between visible and near-infrared portions of the spectrum. This correlates to the electromagnetic domain just beyond the level of emergence associated with an individual atom’s valence shell and the roughly 400–700 nm range of EM wavelengths, in essence multiatomic vibration while a robustly decoherent state prevails. In this theory, if electric current does indeed accelerate at the synapse and through the soma, this would add slightly longer wavelengths to the spectrum. It seems reasonable as a very approximate hypothesis that the spectrum could range from at least 1–10 μm in wavelength. This spectrum is capable of traveling through an aqueous solution at distances of roughly 100 mm to 10 μm, with the range shrinking considerably as wavelength increases (Figure 3). The soma is about 12 cubic micrometers and the synaptic space 1 cubic micrometer, with the currents themselves probably equivalent in volume, so it seems plausible that a persistent field of photonic waves can inundate both. Boosted by maximal reflection from white matter, gray matter may be filled with a substantive light spectrum capable of interacting with molecular arrays and biochemical pathways to form a diversely superpositioned photonic field studded with a wide range of atomic and multiatomic nodes.

Where atoms and molecules involved in the generation of percepts might be most concentrated remains unknown, but protoplasmic astrocytes which are commonly adjacent to the soma and thus have access to hypothesized light fields, with a cytoplasm relatively uncluttered by organelles (Elabbady et al., 2022), are a good candidate, of course in addition to gray matter itself, the soma as well as junctions at which axons and dendrites form synaptic connections. Mounting evidence from studies with paramecia, yeast, onion roots, and even crustaceans substantiates the hypothesis that biophotons of low-intensity travel through cell membranes, affecting functions such as energy production and growth in populations of cells, even when separated by a sizable barrier such as the walls of a glass cuvette (Fels, 2009). A range of wavelengths seem to interact with biochemistry, and all kinds of cellular structures including those of neurons could be built around biophotonic mechanisms.

An exception to the general link between brain hyperthermia and awareness is the visual cortex, where it has been observed with fMRI that tissue temperature decreases by 0.2°C during activation of the neural processing involved (Wang et al., 2014). Some uncertainty exists as to the accuracy of these results, but if valid this suggests molecular structures may exist in parts of the brain to shift the EM radiation spectrum towards shorter wavelengths such as visible light that are less likely to dissipate as the heat of vibrating and rotating chemical bonds. It is intriguing to consider that centers of vision in the brain, probably correlated with the phenomenality of image perception, might generate a light field comparable to the one upon which vertebrate optical mechanisms are based.

Some further categories of mechanism in addition to basic current acceleration seem likely for how spectrums of EM radiation may thicken and assume functional form in the nervous system and brain. Visible, near-infrared, mid-infrared radiation, and perhaps beyond of course must interact with molecules in such a way that wavelengths are modified into a wide variety of vibrational signatures, with all of this dispersing into the sink of somewhat increased temperature during activation as baseline decoherence continually reasserts itself. The electric currents themselves may also rapidly decelerate upon contact with molecular structures to cause braking radiation, and shortened EM wavelengths of relatively low intensity. Whether these processes occur in non-neuronal cells as a result of ion channel activity and additional mechanisms is an interesting topic, barely broached. So how then might this basic substrate of structural integration in the brain, nervous system, and perhaps the wider body give rise to awareness’s percepts, the substance of perception?

## Implications of the coherence field concept for understanding percepts as a physical phenomenon

In the coherence field model, we have thus far formulated, a supervenient EM field drives and orchestrates the behavior of biochemical pathways in the brain, but EM radiation within this material framework is the binding agent which flows around with effective instantaneity to integrate molecular arrays, cells, and tissues at trillions of locations as the vibrational structure of perception. Details of how percepts would form in this manner are undoubtedly complex and, if upheld by further evidence, probably warrant decades of research. But if these theories are accurate, it could provide for some very simple ways to define features of the mind in terms of matter.

This model views percepts, to the extent they arise from electromagnetic properties of tissue, as the emergent organization of atomic nodes within photonic fields, numerous and diverse regions of coherent energy most fundamentally characterized by vibration. The brain is unique because electric currents likely found in all cells are so strong and compact in this organ that a robust EM field is generated which can coordinate the magnetic particles in large swaths of tissue as an individual unit. The brain is thus much more synchronized than the rest of the body. If the hypothesis proves valid, this mechanistic chassis of electrical energy is saturated by EM radiation of a primarily infrared spectral range which interacts with molecules to produce the structural components of the mind, insofar as they arise from the brain, as a variably dense physical field.

Most of our basic sentience—sound, touch, taste, smell, visceral sensations, in essence feel—would essentially be vibrational textures in matter with their shapes, rates of oscillation, and locations determining the quality of experience. Input from specialized sensory apparatuses in the eye, ear, olfactory, gustatory and tactile cells superimposes on fundamentally cognitive textures to render our environment a crisp perceptual world.

Image sensation might be a modification of EM wavelengths within the textural field such that light in the visible range is produced, so that optical inspection and image imagination coevolved into complementary forms. This would explain how we visualize much of what our eyesight takes in without optical stimulation. The visual stream of consciousness is then a complex of visible light and specially adapted cellular structures, while the verbal stream would probably be infrared light and still different biomolecules and cells, together a range of emergent textures induced by the brain and perhaps the wider body. All of this sentience and stream of consciousness converge to constitute the foundational substrate of emotion and thought.

Memory would derive from interaction of this coherent energy field with neural architecture, accounting for how recall cannot be easily pinpointed to any particular process in the brain or body, for it is linked to the interface between field and circuitry at an intracellular level we have not yet penetrated in theory. The relative role of circuitry vs. intracellular biochemistry in memory, synaptic as opposed to intrinsic plasticity, is still the subject of contention (Trettenbrein, 2016; Langille and Brown, 2018), but some form of amalgamation is undoubtedly in play, and the brain’s matter is as photonic and field like as it is molecular. Neural circuitry is built into intricately emergent structures so that synthetic and logic like insights are possible, the environment “making sense” *via* a background of more or less abstract interrelationships rather than just starkly presenting. The self can be defined as a collection of functions that monitor one’s own circuitry and coherence field of radiative/molecular percepts.

This model affords an explication of how a percept’s appearance, we could think of a colored object for instance, is capable of holding stable in our field of vision despite the fact that a dynamic flurry of at least billions of more or less separate atoms participates in producing the image. Field theory implies that particles at the quantum scale are not solid, entirely self-contained, and indivisible units, but rather ripples in an energy field which happen to be especially stable. When particles transition between states and interact, this energy flow is quantized at the subatomic scale, but disjunctions become negligible at the emergent scale of molecular structure, smoothed out into a continuum of flow, just as the components of a robotics plant seem to follow seamless trajectories though zooming in on the process would reveal irregularities and asymmetries of motion. Even with this emergent continuity, partitioning of molecular complexes and biochemical pathways in cellular solution could alone be significant to the appearance of electromagnetic percepts, as robotic machinery at different locations in an assembly line occupies obviously discrepant orientations. But photonic fields, the motions of which are effectively instantaneous at the atomic scale and even the scale of an entire brain, provide an interstitial medium at very basic levels of emergence. Minute regions of disjunction between the organ’s atomic structures as energy maxima are unceasingly integrated by space-saturating waves of EM radiation as energy minima. A coherence field of atoms and EM radiation combined thus veils the fine structure of quantization that obtains at the subatomic scale, shrouding disassociations among energy minima and maxima with an emergent structure which lacks apparent gaps on the scale of biochemical function. From this perspective, the apparent holism of a robotics plant’s physical structure as it conjures an observable scene and the integrated structure of perception insofar as it arises from the brain are parallels in a very real sense: electromagnetic matter’s emergent unity is an “interior” feature as much as an “exterior” one, and if this matter is, in fact, the substance of perception, we should expect physical percepts to evince that permeating unity.

The question of how a coherent field of awareness projects beyond the body can be raised. It must be remembered that coherence is not fundamentally electromagnetic, physiological, or local in the Newtonian sense, and under suitable conditions causality can propagate faster than light. It might be possible for similar mechanisms to those which manifest within the brain and body to conjure beyond physiology, as a hybrid of standing and traveling waves within a medium of infrared light, visible light, and perhaps more energy sources, all interspersed by atomic and molecular nodes with which this energy more or less synchronously vibrates. If an experiment can entangle photons at 3 trillion m/s across a distance of 15 km, any material structure which manipulates the underlying coherence responsible for such entanglement should be capable of similar influence, and the brain could be such a material structure. The coherence field concept may eventually explain why we do not perceive the field of awareness as entirely within our own heads or bodies despite the fact that neural and cellular architecture is required to comprise an organic mind.

Though an EM radiation hypothesis for how matter binds into the substance of perception hangs together well based on what we currently know of physics, it has also been proposed that LFP-based fine structure of the electric field may be the source of percepts. Any region of this field is of course composed of numerous superpositioned frequencies which can be decomposed by a Fourier transform in similarity to EM radiation, producing the familiar EEG readouts. The question is whether this reaches enough complexity to be the sole seat of perception.

As an example, we can estimate the maximum intricacy of an electric field consciousness. If we assume percepts are superpositions delimited by phase-locking, of which the basic unit is some constitutive portion of an LFP, the most complex and differentiated consciousness possible for a human would plausibly consist in neural networks of on average a hundred phase-locked neurons each, blending into both a background of slower waves and some kind of roving, semi-stable density of relatively homogeneous frequency that temporarily mingles with a variety of more local oscillations to produce experiential awareness. If phase-locking determines the boundaries of a percept, and the brain contains approximately 80 billion neurons making 100 trillion connections, each neuron would contribute to on average around 1,250 different percepts at most. This hypothetical consciousness would support 800 million simultaneous percepts and 1 trillion percepts total. But human olfaction detects more than a trillion scents (Bushdid et al., 2014), and this is one of our least acute sensory modalities, in addition to being localized within small portions of the brain. The range of variation in sounds and images far exceeds olfaction. Overall oscillation patterns within one of these minimum phase-locked assemblies may involve a continuum of relativities rather than simply being a steady state, on or off phenomenon, doing double duty in the formation of multiple percepts, so within any particular neural network the spectrum of percepts might be much greater, though the level of differentiation must at some point prove discrete, constrained by an LFP’s degrees of freedom. We must also consider that much of the brain may not be sufficiently phased for producing emergent organization conducive to percepts of this type, so the possible quantity of percepts would likely be much less than the maximum. Of course, pending further research, room for doubt exists as to whether an LFP-based model alone is capable of accounting for the full gamut of percepts.

It is also uncertain how an LFP-based model can explain the nonlocality of consciousness. At this point, extrasensory perception is fairly well-established scientifically, since it has been demonstrated that humans can communicate, locate archaeological sites, etc. through ESP (Schwartz et al., 2022). Science is making rapid progress in its capacity to model faster than light entanglement between photonic fields, an action at a distance which is canonical to quantum physics. Though advances are being made in finding ways to empirically verify the mechanisms of group consciousness phenomena, in essence, brain wave entrainment and synchronization between multiple individuals *via* some behavior-linked mechanism, research is in its early stages (Young et al., 2022). Though brain waves are of course integral to the presentation of all conscious phenomena, we presently have much less cause for attributing ESP to mediation by LFPs and emergent flow shapes in the electric field than entanglement dynamics of the EM radiation they contain.

If we add EM radiation to the electric field model, this massively increases the diversity available to perceptual mechanisms, from maximums of roughly a few trillion superpositioning LFP subunits to at least hundreds of trillions of possible locations where photonic fields, variously superpositioned on scales resembling spectra in the external environment, can cohere with atoms and molecules to assume functional form. These photonic fields which would radiate with effective instantaneity in the brain may get locked in as emergent structure during neural activation, with the signature of this light modulation mechanism being temperature variation. To the extent that a region of the brain is especially saturated by synchronizing mechanisms such as phase-locking, as seems to be the case in processes of experiential awareness, the effects of photonic fields would simultaneously become more pervading. The LFP-based model and photonics model are thus complementary, for if research proves that EM radiation plays a functional role, this is simply an intrinsic aspect of the electric field’s fine structure as it oscillates and flows.

Cross talk between neural regions within the 100 ms temporal window during which perceptual binding occurs would be greatly enhanced by the light speed effect of large-scale oscillations in the brain’s electric field on LFP oscillons, and some models indicate that this type of modulated superposition amid oscillators is necessitated in a process such as percept binding (Kraikivski, 2022). Line of sight issues must limit the intricacy of interaction between the brain’s somewhat partitioned electric field and microscale oscillons, which we of course observe, but if EM radiation can be included this proliferates the fine structure of modulated superposition by orders of magnitude. If justified by a continuing train of evidence, the hybrid electric/radiative field model makes neuroscience and quantum physics natural collaborators, for brain/body and nonlocal phenomena of consciousness may yield to a single explanation rather easily provided EM radiation is a binding agent for the physiology and more generally the matter of perception in addition to the physical environment as a whole.

The mechanism by which brain matter contributes to forming the substance of percepts is proposed by this article as starting with a sustained acceleration of electric current between centers of ion concentration, modifying the spectrum of EM radiation (primarily infrared and more rarely visible light) while increasing its quantity. This proceeds to modulation *via* a cascade of light/molecular interactions, ending in a temperature increase when decoherence thermally dissipates the additional energy as biochemical vibration and infrared radiation. If current acceleration is steady enough, the electromagnetic energy that results can maintain intracellular coherence fields, and likely also intercellular coherence fields due to the transmission of EM radiation through cell membranes. But this mechanism might preclude coherence fields spread through complexes of axons because myelin reflects any infrared or visible radiation from intracellular currents back into the neuron.

An alternate mechanism not discussed with much depth in this article is the manipulation of molecular arrays through EM field permutations that can originate from electron and ionic currents. Modified vibration of molecules might then induce a separate route to cascades of modulated light/molecular interaction, also thermally dissipating as biochemical vibration and infrared radiation due to decoherence. The range at which this mechanism can modulate a coherence field depends on the density and location of affected atoms and molecules, but could conceivably transcend the limitation that myelin imposes on axons and adjacent extracellular space because of transmembrane influence, expanding the perceptual field to brain matter in its entirety. Further effects along these lines are probably transmitted *via* emergent electric wave oscillations and flow spread through macroscopic portions of the brain, synchronously morphing LFP/neural complexes, current-field patterns, and the coherence fields of cellular structure in a top-down way to enact larger-scale perceptual integration. It is well-established that endogenous electric fields affect orientation, migration, adhesion, proliferation and differentiation among and within cells (Cassela et al., 2021). Alteration to a cell’s molecules *via* electric fields is finding application in cancer treatment as TTFields (tumor treating fields; Tuszynski et al., 2016), wound healing, and the modification of developmental processes (Cassela et al., 2021). Numerous research angles already identify electric field properties as integral to workings of the cell, and once the investigation has matured enough to comprehensively assess the dynamics of varying electric field strength and distribution along with the undulating EM radiation within these fields, any electric field/molecular routes to coherence field modulation should be modelable.

A third possibility is that so-called nonlocal properties of the brain’s coherence field facilitate entanglements *via* EM radiation and through this route modulate cascades of light/molecular interaction. Mechanisms of this type could pervade the brain, exacting an extremely holistic effect upon electromagnetism, with the vibrational and radiative consequences being at this stage unknown and fairly unpredictable. We cannot rule out the potential for modification of EM radiation and molecular vibrations into many different forms than would be predicted in association with electric currents or LFPs.

The question then is how we are to derive definitive models of these mechanisms and their comparative role in the brain. It is very early in the research agenda, so analysis of the correlation between electric currents, EM fields, and the modulation of EM radiation must probably take place outside a neural context, with stripped down experimental designs restricting mass, velocities, volumes, concentrations, temperature, etc. to a small, inorganic set of variables, gradually building our facility in parameterizing how light and electrons in motion should interact within biological systems. Developing techniques for measuring the emission of EM radiation within and from neurons would help tremendously, an investigation which can probably be extended to the entire body. Physics will of course continue to construct more incisive models of nonlocality, and this phenomenon’s intersection with brain function can be complemented by psychology of nonlocality. If perception is a physical field at least partially manifesting as an electromagnetic matter of the brain, continuing revelations of physics will surely mesh well with predictions of neuroscience and are valuable in and of themselves. We have decent prospects for a comprehensive physical theory of percepts.

## Conclusion: a coherence field theory of consciousness

So in summary, our increasingly sophisticated understanding of matter at the subatomic scale suggests that electric currents in neurons are driven by states of quantum coherence which occur within an aqueous solution of ions. The baseline condition is for aqueous solution to be maximally decoherent due to nanoscale shifting among huge quantities of polar constituents, but when charge disparity is induced between large enough centers of ion concentration, a coherent current flows from higher electron density or more negative charge to lower electron density or more positive charge. Neural anatomy is built around this dynamic, with ion channels modulating the flow of electron current to transmit signals between nodes at a significant fraction of light speed. The electric current mechanisms of individual neurons may provide a deep explanation for why the electric fields of neurons oscillate, why axons contain juxtaparanodes, why nodes are spaced as they are in proportion to neuron diameter, and a host of further observations. It is not a stretch to claim that the coherence current concept may tie all we have discovered of neural anatomy into a comprehensive model of intraneural function.

The source of intercellular LFPs and phase-locking of emergent brain waves with neural networks is more in the realm of speculation, but a computer model based around ionic current flow through membrane channels alongside analysis of discrete synapses holds promise for making inroads on this front, while additional mechanisms may obtain. The brain’s electric field increasingly appears to be a central factor in consciousness’ integration rather than an epiphenomenon of neuronal activity, and ongoing research into macroscopic oscillation and flow patterns as coordinated with tissue should continue to reveal more about the organ’s functional organization.

Neural tissue is more closely correlated with temperature fluctuations than is an anatomy of comparable locations such as the brain’s arteriole blood vessels. This thermal energy is instantiated as vibrating molecules integrated with fields of EM radiation which in a physiological context peak within the infrared range of the spectrum. The hypothesis is that these vibrational and wavelength signatures are not a mere waste byproduct, but prove intrinsic to the electromagnetic matter as it binds into expanses of coherent structure modulated to produce many of the first-person experience’s basic features, what I have called percepts. A potential mechanism linking the coherence currents of a neural solution to modulation of these coherence fields has been discussed in depth, and additional mechanisms are possible. In this model, brain processes which generate states of coherence are an essential facet of the organ’s material structure such as the visual system’s retinotopic maps, and these states coalesce as basic constituents of perception on the intracellular and perhaps intercellular scale. The contents of experiential awareness, attention, and will are then to a profound degree emergent from properties of coherence evinced by electromagnetism.

The primacy of vibration and wavelength to these ideas aligns closely with General Resonance Theory, and it seems promising that resonant phenomena identified at the nanoscale will reveal themselves to be important aspects of first-person perception. Experimentally verifying the main hypotheses of coherence field theory would constitute major progress in addressing the “quantum question” Hunt and Schooler have outlined. This pushes beyond statistical formulations of quantum mechanics to begin constructing a phenomenology of matter suitable for full integration of physics with the life sciences. Perhaps a scientific solution to the mind/body problem is just around the corner.

## Data availability statement

The original contributions presented in the study are included in the article, further inquiries can be directed to the corresponding author.

## Ethics statement

Ethical review and approval was not required for animal studies because research cited was performed by neuroscientists who met the ethical standards necessary to publish. Ethical review and approval was not required for studies on human participants in accordance with the local legislation and institutional requirements. Written informed consent for participation was not required for this study in accordance with the national legislation and the institutional requirements.

## Author contributions

EB has exclusively developed, analyzed, and authored the contribution of coherence field theory to a model of consciousness: electric currents, EM fields, and EM radiation in the brain.
